# Enigmatic roles of *Vibrio cholerae* hemolysin/cytolysin in the bacterial pathogenesis and host-pathogen interactions

**DOI:** 10.1128/jb.00248-25

**Published:** 2025-07-28

**Authors:** Mahendra Singh, Sindhoora Puravankara, Arunika Mukhopadhaya, Kausik Chattopadhyay

**Affiliations:** 1 Department of Biological Sciences, Indian Institute of Science Education and Research Mohali, SAS Nagar, Manauli, Mohali, Punjab, India; Geisel School of Medicine at Dartmouth, New Hampshire, USA

**Keywords:** *Vibrio cholerae*, pore-forming toxins, *Vibrio cholerae* cytolysin, hemolysins, exotoxins

## Abstract

*Vibrio cholerae* cytolysin (VCC; also known as *V. cholerae* hemolysin) is a β-barrel pore-forming toxin (β-PFT) secreted by the cholera pathogen *V. cholerae*. VCC acts to disrupt the selective permeability barrier function of the target cell membranes. Monomeric VCC molecules bind to and form heptameric transmembrane water-filled pores or channels in the lipid bilayer of the membranes, thus resulting in colloid-osmotic lysis of the target cells. Apart from its pore-forming function, VCC can activate an array of signaling cascades leading to the diverse responses that include programmed cell death, autophagy, inflammation, etc. VCC has been studied extensively focusing on the biochemical, biophysical, and structural aspects of the pore-formation mechanism. In contrast, the mechanistic basis of the VCC-mediated programmed cellular responses and their implications for bacterial pathogenesis and host-pathogen interaction processes have received less attention in the past. However, more recent studies have highlighted the crucial importance of the pore-formation-independent cellular responses for the *V. cholerae* pathogenesis process. Nevertheless, several questions regarding the pathophysiological contributions of VCC remain unanswered. In this minireview, we provide a brief account of the historical perspective of VCC in the context of *V. cholerae* pandemics, its pore-formation mechanism, and distinct cellular responses that could be evoked by this exotoxin in its target host cells. We also highlight some of the unanswered questions regarding its pathophysiological attributes and their potential contributions during the bacterial infection.

## INTRODUCTION

Cholera is an acute, severely dehydrating diarrheal disease caused by the Gram-negative bacterial pathogen *Vibrio cholerae*. Cholera persists in the underdeveloped countries, mostly due to the lack of clean drinking water and unhygienic living conditions. Cholera infection leads to acute symptoms like severe diarrhea and vomiting, causing hypovolemic shock, and can even lead to death if left untreated. Since 1817, seven cholera pandemics have been reported, and the seventh one, starting from 1961, is continuing to date. As per the recent World Health Organization (WHO) document, ~824,479 cholera cases and ~5,900 deaths have been reported from 31 countries across five WHO regions from January 2023 to March 2024. In January 2023, WHO classified the global re-emergence of cholera as a grade 3 emergency (https://www.who.int/publications/m/item/multi-country-outbreak-of-cholera--external-situation-report--13---17-april-2024). Additionally, as notifying the cholera cases to WHO is not mandatory, it remains heavily underreported, indicating a much more devastating situation (1). Whatsoever, these reports indicate that cholera is one of the main causes of morbidity and mortality in several underdeveloped and developing countries, thus making it critically important for public healthcare studies.

*V. cholerae* is classified into more than 200 serogroups depending on the structure of the O-antigen of lipopolysaccharide present on its outer membrane. A subset of the strains from serogroups O1 and O139 is considered to be virulent due to their ability to produce cholera toxin (CT) and is, therefore, associated with the epidemics and pandemics of cholera. Serogroups non-O1 and non-O139 are unable to produce CT but can cause small gastroenteritis outbreaks, wound infections, and sporadic cases of bacteremia (2–4). Furthermore, the O1 serogroup is subdivided into three serotypes, Ogawa, Inaba, and Hikojima, and two biotypes, classical and El Tor. Another serogroup has emerged during the epidemics from eastern India and Bangladesh and has been designated as “*V. cholerae* O139 Bengal,” which appears to be a hybrid of O1 and non-O1 strains. This strain is indistinguishable from the El Tor and classical strains in terms of virulence characteristics, such as the presence of cholera enterotoxin and toxin-coregulated pilus (TCP) (5). Recently, the number of non-O1 and non-O139 infections has increased worldwide, ranging from sporadic infection cases to local epidemics. Although not severe and mostly self-limiting, sometimes these infections have also been reported to cause severe gastroenteritis and even cholera-like symptoms. It also remains a concern that additional potential virulence agents, apart from CT, are possibly carried in the arsenal of these strains and can contribute to the pathophysiological outcome.

*V. cholerae* genome is comprised of two chromosomes: chromosome 1 and chromosome 2. Chromosome 1 contains all the major virulence factors, including CT and TCP (6). CT is a classical AB-type toxin and contains a larger “A” subunit and five “B” subunits. The A subunit of CT leads to the ADP-ribosylation of G-protein, resulting in the continuous activation of adenylate cyclase and an increased level of cyclic adenosine monophosphate (cAMP) production in the target cells. This leads to the rapid efflux of chloride ions combined with sodium ion influx, resulting in massive water efflux from the intestinal cells, causing severe diarrhea. Apart from these major virulence factors, accessory toxins such as multifunctional auto-processing repeats-in-toxin (MARTX) and zonula occludens toxin (ZOT) are also secreted by *V. cholerae*. MARTX is secreted through the type I secretion system and forms pores in the plasma membrane, leading to the translocation of multiple toxin-effector domains into the intestinal cells. This toxin provides a protective role from the neutrophil-mediated clearance of the bacteria, contributing to enhanced bacterial colonization during the early stages of infection (7–9). Another toxin, ZOT, is reported to modulate the structure of the actin microfilaments, affecting the permeability of intestinal tight junctions that, in turn, results in the paracellular passage of large molecules (10). The accessory cholera enterotoxin (ACE) is an integral membrane protein that can alter ion transport and cause fluid accumulation in the rabbit ileal loop. Before the stimulation of the slow-acting CT, ACE may contribute to the intestinal secretion and diarrhea by activating the calcium-dependent Cl^−^/HCO^3−^ symporters, which leads to the influx of extracellular Ca^2+^ (11). Apart from the above-mentioned toxins, *V. cholerae* is reported to produce a number of other toxins, such as non-agglutinable heat-stable enterotoxin (NAG-ST) and sodium channel inhibitor (12). NAG-ST is produced by some strains of *V. cholerae* non-O1, which shares 50% homology with the heat-stable toxin (ST) of enterotoxigenic *Escherichia coli* (13, 14), and has been shown to cause diarrhea and gastroenteritis (15). In some of the strains of *V. cholerae* non-O1, a gene encoding thermostable direct hemolysin-like toxins has also been found (16).

Another important virulence factor produced by *V. cholerae* is a pore-forming toxin (PFT), designated as *V. cholerae* cytolysin/hemolysin (VCC). VCC exhibits potent enterotoxicity and fluid accumulation in the rabbit ileal loop model of the diarrheal disease (17, 18). As a hemolysin, VCC causes lysis of erythrocytes from various animal species (19). The exact contribution of VCC as one of the major virulence factors of *V. cholerae* has remained an enigma. Nevertheless, VCC is always appreciated as one of the most important toxins produced by *V. cholerae,* particularly by the strains that are unable to produce the major toxin CT (17). Structurally, VCC belongs to the family of β-PFTs (20–23). Consistent with the β-PFT mode of action, VCC possesses the remarkable ability to form oligomeric pores in the plasma membranes. These pores act as non-specific diffusion channels, allowing free passage of ions, water, and small molecules. This pore-forming function disrupts the selective permeability barrier function of the target cells’ plasma membranes, leading to the colloid-osmotic lysis of the cells. VCC is also reported to induce a plethora of intracellular signaling cascades leading to cell death and/or the generation of inflammatory/immune responses (17, 24–26). This minireview presents an overview of our current understanding regarding the pathophysiological functions of VCC, and their implications for the *V. cholerae* pathogenesis and host-pathogen interaction processes. We also aim to highlight some of the crucial, yet unanswered questions regarding the pathophysiological implications of the VCC mode of action.

## BRIEF HISTORY OF VCC: A β-BARREL PORE-FORMING TOXIN

As reported in a number of past studies, certain *V. cholerae* strains isolated from patients with cholera-like symptoms have been found to be devoid of CT, the major virulence factor that is otherwise responsible for the pathophysiological manifestations of cholera. However, the virulence factors leading to these symptoms remained unidentified for a long time. Eventually, one putative factor has been identified from the bacterial cultures as an extracellular cytolytic protein and has been designated as the *V. cholerae* hemolysin, owing to its potent hemolytic activity. This protein has been shown to display the lytic effect toward the erythrocytes and cytotoxic activity against a wide spectrum of mammalian cells (27, 28). Based on such observations, this hemolysin has been considered to act as another potential virulence factor, which might function as a major toxin in the *V. cholerae* strains lacking CT. This cytolytic/cytotoxic toxin is produced by the *V. cholerae* El Tor O1 and non-O1/non-O139 strains, and is also known as the El Tor hemolysin (HlyA) or VCC.

VCC is encoded by the *hlyA* gene that encodes for an ~82 kDa precursor protein, termed “pre-pro-VCC.” Pre-pro-VCC consists of an N-terminal signal peptide, along with an N-terminal ~14 kDa pro-domain, followed by the C-terminal mature VCC protein of ~65 kDa molecular mass (24). Generation of the mature form of VCC involves a two-step process. First, the conversion of pre-pro-VCC into the inactive “pro-VCC” takes place by the cleavage of the signal peptide during the transport of the polypeptide through the inner membrane of the bacterium. In the second step, the pro-domain of the secreted inactive protein is cleaved by the extracellular metalloprotease, such as HA/protease (29). This conversion can also be achieved *in vitro* by employing proteases such as trypsin, chymotrypsin, and subtilisin (22). Additionally, certain cell surface proteases have also been shown to perform this maturation of VCC from the inactive precursor state. The pro-domain of VCC functions as an intramolecular chaperone that assists in the proper folding of the toxin into the functionally active form. The pro-domain also plays a crucial role in the transport of the protein from the periplasmic space across the bacterial outer membrane into the extracellular medium (30).

**Fig 1:**
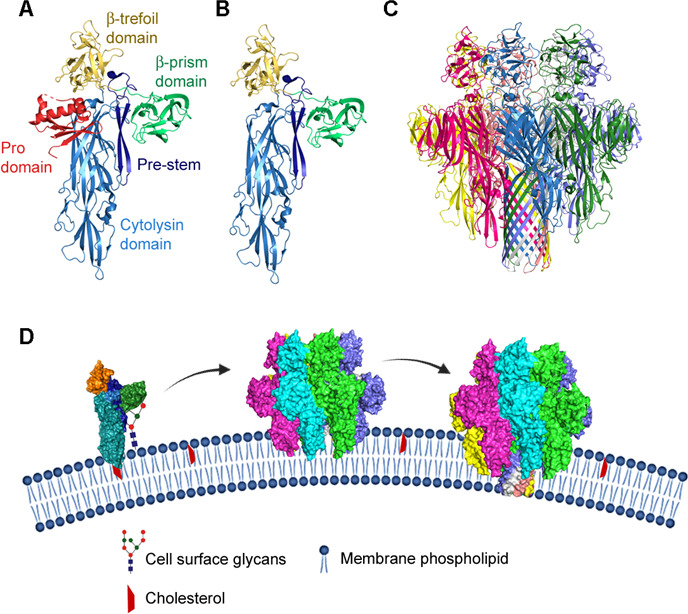
VCC is a β-barrel pore-forming toxin. (**A**) Structural model of pro-VCC monomer showing different domains and motifs of the toxin (based on PDB ID: 1XEZ). (**B**) Mature VCC monomer without the pro-domain. (**C**) Structural model of the heptameric pore-complex of VCC. Protomeric subunits of the complex are shown in different colors (based on PDB ID: 3O44). (**D**) Pore-formation mechanism of VCC includes three distinct steps. First, the mature monomeric VCC molecules bind to the target cell membranes. Membrane-bound VCC molecules then oligomerize to form transient heptameric pre-pore assemblies on the target membrane. The pore-forming pre-stem motif(s) from each protomer extend and insert into the membrane, forming the functional transmembrane pore complexes and completing the process of pore formation.

The mature and active form of VCC has been shown to lyse erythrocytes and various eukaryotic cells by forming heptameric transmembrane pores of 1–2 nm diameter (24, 31). The crystal structures of the monomeric pro-VCC and heptameric VCC pore complex have revealed structural features of the toxin in both the soluble precursor state and membrane-embedded pore state (Fig. 1A through C) (21, 22). These structural models have also highlighted the intricate details of the structural/assembly changes associated with the VCC pore-formation process. Mature VCC is comprised of three major structural domains: the cytolysin domain that constitutes the central scaffold of the protein, and two lectin-like domains termed the β-trefoil domain and the β-prism domain. The cytolysin domain is the central domain of VCC that harbors the pore-forming motif, which contributes to the formation of the transmembrane β-barrel scaffold of the pore structure. This pore-forming motif remains packed (in the form of a so-called “pre-stem” motif) against the core cytolysin domain in the soluble monomeric state of the toxin (21, 22). Upon binding to the target cell membranes, VCC undergoes specific conformational/assembly changes, and the monomeric units come together to form the transient metastable intermediate assemblies that are commonly defined as the pre-pore(s). The pore-forming pre-stem motif(s) from the seven protomers then extend and insert into the lipid bilayer of the target membranes to attain the “stem” configuration, forming the functional transmembrane β-barrel pore (Fig. 1D) (32, 33).

The exact mechanism by which VCC binds to the target cell membranes remains an enigma. VCC shows specific binding to the membrane phospholipids. In addition, the presence of cholesterol and sphingolipids in the membranes seems to facilitate membrane-binding and pore formation. Furthermore, the specific interactions of VCC with the putative glycan receptors in the target cell membrane have also been proposed to assist in the membrane-binding and pore-formation process of the toxin (34–36). It is intriguing that VCC employs multi-faceted interaction mechanisms to establish its association with the target cells. Interestingly, VCC shows different extents of hemolytic activity against the erythrocytes from different species. For example, rabbit erythrocytes are much more susceptible to the VCC-induced lysis, as compared to the human erythrocytes (19, 37, 38). Furthermore, VCC shows significantly reduced pore-forming ability in the liposomes (lipid vesicles) composed of lipid components only, as compared to that in the biomembranes of erythrocytes. These observations indicate the presence of potential receptor(s) (particularly in the biomembranes of rabbit erythrocytes) that could augment the efficacy of VCC functioning. However, the exact identity of such receptor(s) remains unknown. Apart from that, membrane fluidity, surface charge, and the presence of protective membrane-associated proteins may also contribute to the species-specific sensitivity.

Owing to its potent membrane-damaging pore-forming activity, VCC induces lysis of erythrocytes and killing of a wide variety of nucleated cells that include epithelial cells as well as the cells of the immune system. Apart from inducing pore-formation-mediated cell death, VCC is known to trigger programmed cytotoxic responses, such as apoptosis in the target cells (24). VCC has also been shown to exhibit potent enterotoxicity in the ligated rabbit ileal loop model of diarrheal disease (18). These properties mark VCC as a potent virulence factor that may assist in the pathogenesis process of *V. cholerae*.

The pathogenicity of the *V. cholerae* strains is mostly due to the production of highly virulent factors such as CT. However, the strains that lack CT are frequently isolated from aquatic environments and are reported to cause sporadic outbreaks of watery diarrhea and inflammatory enterocolitis. These reports, combined with *hlyA-*deletion studies (deletion of the gene coding for VCC), indicate the potential involvement of VCC in inducing diarrheal symptoms, thus holding an important role of VCC in the *V. cholerae* pathogenesis (17, 39). Additionally, VCC is capable of inducing chloride ion efflux from the intestinal epithelium, providing further evidence that it can act as a major diarrheagenic factor, especially in strains lacking CT production (39). Altogether, VCC has been studied to uncover its potential role in *V. cholerae* pathogenesis. However, the exact role of VCC in the cholera pathogenesis remains obscure.

## STRUCTURE-FUNCTION STUDIES ON THE PORE-FORMATION MECHANISM OF VCC

The pore-formation mechanism of VCC has been studied in detail. It has been elucidated that the monomeric VCC, in its mature form (without the pro-domain), binds to the target membranes in a reversible manner and then oligomerizes to form the heptameric assemblies on the target cells. These assemblies are transient and metastable and are commonly designated as the pre-pore assemblies. These pre-pores then convert into the final and functional pore-complex by inserting the membrane-penetrating pre-stem motifs into the target lipid bilayer (32, 33, 40, 41).

The crystal structures of the VCC monomer and heptameric pore have been elucidated (21, 22). Recent studies have also reported the cryo-EM structures of the VCC pore complex in the lipid membranes (42). These structural studies have established that VCC forms heptameric pores of homogeneous stoichiometry and size. Furthermore, these structural studies, along with extensive biochemical and biophysical studies, have provided critical information regarding the intricate mechanistic details of the VCC pore-formation mechanism, deconvoluting the process into three distinct, and possibly sequential steps. First, the monomeric VCC molecules interact with the target membranes. These interactions govern the binding of the toxin molecules to the target cells. Not only the composition of the target membranes, but also the physicochemical parameters of the membrane environment, also regulate the binding efficacy of VCC (31, 43). Overall global amphipathicity of the VCC molecular architecture has been shown to be one of the early driving impetuses for the toxin monomers to partition into the amphipathic phase of the membrane lipid bilayer environment (44). However, VCC establishes more specific interactions with the membrane phospholipid headgroups that act to regulate the binding and post-binding steps of the pore-formation mechanism. VCC has been shown to employ specific structural motifs to recognize and bind to the membrane phospholipid headgroups (34, 35, 38). Pore formation by VCC has been found to be more efficient in the Asolectin-based liposomes compared to that in the phosphatidylcholine-based liposomes. This observation indicates a preference of VCC for the specific phospholipid composition of the target membranes. In addition to the presence of specific phospholipids, cholesterol has been shown to facilitate the membrane interaction ability of VCC, particularly in the absence of the non-lipid-dependent interactions. Also, a sufficient level of cholesterol is required for the functional pore formation, converting oligomeric pre-pores into functional pores. VCC tends to get partitioned into the cholesterol-rich membrane micro-domains, or lipid rafts, in the biomembranes of human erythrocytes. Interaction of VCC with cholesterol is considered to be essential for the pore-forming activity of the toxin. In addition to the specific membrane phospholipids and cholesterol, the ceramide moiety of the sphingolipids has been shown to promote the pore-forming activity of VCC, presumably by modulating the physical properties of the target membrane, by exposing the membrane-embedded cholesterol for the efficient toxin interaction (34, 35, 45).

Previous studies have proposed the involvement of potential proteinaceous and/or non-proteinaceous putative receptors for VCC. Sialoglycoprotein glycophorin and specific cell surface glycans have been shown to enhance the efficiency of the pore-formation process of VCC in the erythrocyte membranes (44). The β-prism lectin-like domain of VCC functions as the structural module that contributes toward the ability of the protein to interact with the β1-galactosyl-terminated complex glycoconjugates present on the target cell surface (36, 46). It has also been speculated that the lectin activity of the β-prism domain may act as a triggering switch for the structural rearrangements that are crucial for the functional oligomeric pore-formation process of VCC (36). Interestingly, the other lectin-like domain of VCC, the β-trefoil domain, may not be engaged in any glycan-dependent interactions; rather, it is considered to maintain the stability and folding of the toxin molecule (47). However, the true function of the β-trefoil domain remains unknown to date.

In the later steps of the pore-formation process, the membrane-bound monomeric molecules of VCC self-associate and form the heptameric assemblies. Previous studies have identified a number of residues that might be implicated in the monomer-monomer interactions responsible for the formation of these assemblies. Most of these residues are present in the cytolysin domain, such as Asp-214, Arg-330, and Phe-581. Mutational studies involving these residues suggest their important role in governing the oligomerization of the membrane-bound VCC molecules (21, 48). A number of structural and biochemical studies have confirmed the existence of the transient pre-pore intermediate state(s) in the VCC pore-formation. These intermediate assemblies, before converting into the functional pores, remain in the form of membrane-bound oligomeric states (32, 33). Subsequently, the pore-forming pre-stem motif(s) from each of the toxin protomers extend and insert into the lipid bilayer, forming the functional β-barrel transmembrane pore (Fig. 1D). Formation of the oligomeric assembly structure by VCC is critically required for the insertion of the pore-forming motif(s). Additionally, the pore-forming process involves a number of conformational changes in the protein structure that are crucial for the functional pore formation (21, 22, 32). Despite extensive efforts, the pore-formation mechanism of VCC and its regulation are not completely understood.

## CYTOTOXIC RESPONSES INDUCED BY VCC IN THE TARGET NUCLEATED CELLS

VCC not only exhibits hemolytic activity against the erythrocytes, but it also triggers potent cell death responses in a wide variety of nucleated mammalian cells (17, 49, 50). Owing to its potent membrane-damaging pore-forming activity, it is not surprising that VCC would kill the target eukaryotic cells. However, in addition to the pore-formation-induced direct cell death, VCC induces a wide spectrum of effects in the target nucleated cells that altogether can contribute to the cytotoxicity, including apoptotic programmed cell death responses (17). Numerous studies have explored the effects of VCC on the target cells that ultimately lead to apoptotic cell death and other responses. The primary infection site of *V. cholerae* is the intestine. Consistent with this, VCC has been shown to affect the intestinal cells in multiple ways. For instance, the rounding of the intestinal epithelial cells has been observed following the VCC treatment (49). It has been shown that VCC causes ATP depletion in the monolayers of epithelial cells in a dose-dependent manner (51). VCC has been shown to induce apoptotic activity in human intestinal cells (17). Studies on HeLa cells have revealed that VCC treatment causes cell vacuolation (52). Similarly, Vero cells exposed to *V. cholerae* culture media exhibit cytotoxicity and vacuolation that are primarily attributed to the activity of VCC (53). VCC-induced vacuolation is a multi-step process involving initial pore formation and ion imbalance, followed by trafficking through the endo-lysosomal system, and possibly autophagic engagement in certain cell types (54, 55). These cytotoxic responses may vary depending on the toxin concentration and different cell lines exhibiting varying sensitivities. Compared to *Helicobacter pylori* VacA, a potent cell vacuolating toxin, VCC has been reported to induce vacuolation in HeLa, Vero, and A431 cells at a much lower concentration range, indicating the potent cytotoxic properties of VCC (54). Additionally, VCC-mediated vacuolation also depends on the autophagic pathways that are reported to act as a protective strategy from VCC-mediated membrane damage (55). Indeed, VCC exposure has been reported to be lethal for most of the cell types studied so far (54). Intestinal cells, such as T84, are close to the primary target cells for VCC in a natural infection scenario. VCC at higher concentrations leads to progressive damage to the monolayers of these epithelial cells, killing the cells eventually, with extensive vacuolation (54). Caspase-dependent activation of apoptotic pathways also contributes to the cytotoxic responses by VCC. Activation of this programmed cell death pathway involves different caspases, such as caspase-3 and 9, in various cell types, including intestinal epithelial cells, macrophages, and B-cells (50, 56, 17).

As a PFT, VCC forms oligomeric pores in the target cell membranes that can induce colloid-osmotic lysis of the cells, presumably due to the unrestricted flow of water and ions across the plasma membrane. Primarily, potassium and calcium ions are the major ones that get transported across the plasma membrane. Earlier studies have shown that low doses of VCC can lead to the efflux of intracellular potassium with no calcium influx. Low doses of VCC have been reported to induce rapid leakage of cellular potassium ions in the intestinal epithelial cells, followed by ATP depletion (49). The loss of potassium appears to occur through the pores formed by VCC rather than through the severely damaged membrane (49). Many other PFTs, such as hemolysin secreted by *E. coli* and thermostable direct hemolysin secreted by *V. parahemolyticus*, have been shown to form Ca^2+^-permeable pores that can alter the calcium homeostasis in the nucleated cells (57, 58). In contrast to other PFTs, VCC pores appear to be mostly impermeable to the calcium ions (49). Even though VCC pores are impermeable to calcium, VCC has been shown to increase the intracellular calcium levels to a moderate level in mast cells and intestinal epithelial cells (59, 60). Previous studies have shown the importance of calcium in the immune response generation against VCC in the mouse bone marrow-derived mast cells (60).

## VCC-INDUCED PROGRAMMED CELL DEATH AND THE ROLE OF PORE FORMATION IN CYTOTOXICITY

In addition to the pore-formation-mediated cellular damage, VCC has been shown to exert cell killing through many other pathways. One such pathway is programmed cell death or apoptosis, in which cells undergo a series of programmed events, ultimately leading to cell death. Earlier reports have demonstrated that VCC exerts lysis of the intestinal cells, such as Caco-2, at higher toxin concentrations, whereas at lower concentrations, it induces vacuolating activity (52, 54, 61). In model organisms such as *Caenorhabditis elegans,* VCC has been shown to induce lethality, tissue damage in the form of vacuoles, constriction along the intestine, and even developmental delay (62). VCC has been shown to induce DNA fragmentation in the target cells, a signature feature of apoptosis along with caspase-3 activation (17). Additionally, VCC has been shown to cause an increase in the sub-diploid DNA content in the cells, including Caco-2, J774, and COS-7 cells, which is a characteristic feature of apoptosis (17). Similarly, it has been observed that VCC can cause ultrastructural changes such as chromatin condensation, nuclear fragmentation, and plasma membrane blebbing, providing further evidence to confirm the induction of apoptosis-like cell death upon exposure to the toxin (17, 59).

**Fig 2:**
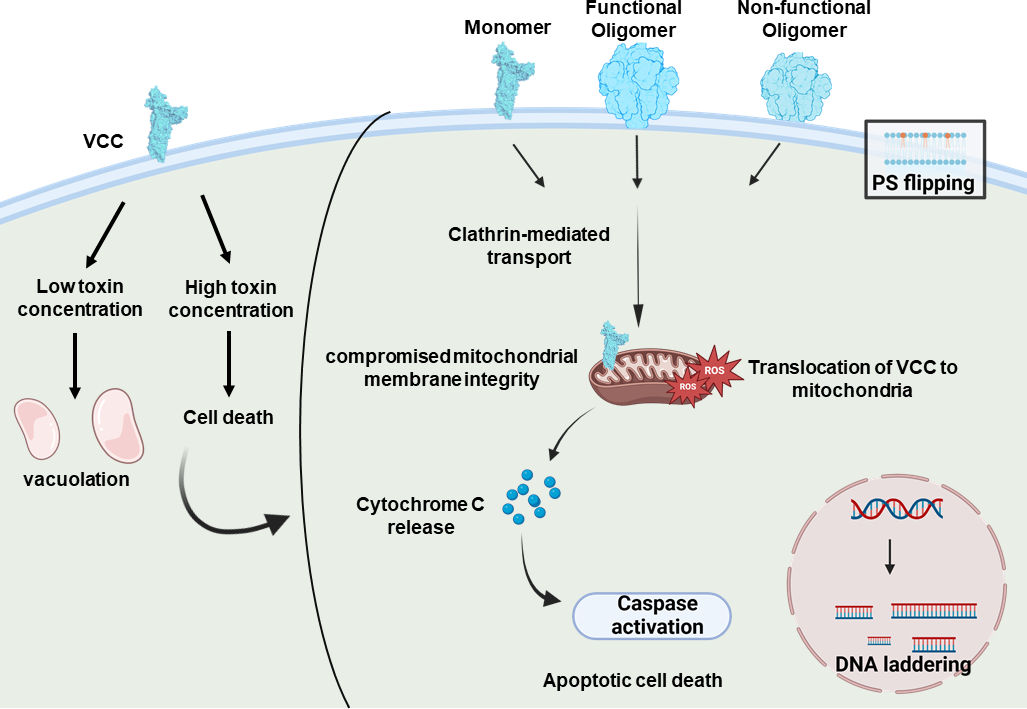
VCC-induced programmed cell death. VCC can affect the target cells in various ways depending on the toxin concentration. At lower concentrations, it can activate vacuolation in cells, and at higher concentrations, VCC has been shown to induce cell death. The active form of VCC generates functional transmembrane pores and induces all the hallmark features of apoptosis. Mutants of VCC that lack the ability to form a functional pore revealed that even in the absence of pore-forming capability, VCC can activate programmed cell death pathways. VCC mutant that is unable to oligomerize remains arrested in a membrane-bound monomeric form, and another mutant that lacks the pre-stem motif essential for membrane insertion, resulting in the formation of inactive, non-functional oligomers. Despite these deficiencies, VCC can translocate into the mitochondria via a clathrin-mediated endocytic pathway, leading to compromised mitochondrial membrane integrity. The loss of mitochondrial membrane potential causes the release of cytochrome C, followed by caspase activation, DNA fragmentation, and phosphatidylserine flipping. These findings indicate that the ability of VCC to induce cytotoxicity does not rely solely on its pore-forming capability.

Cells undergoing apoptosis exhibit distinct morphological characteristics along with certain alterations at the molecular level. Intestinal epithelial cells treated with VCC demonstrate significant shrinkage in the cellular volume and increased granularity, characteristic morphological features associated with apoptosis (59). Activation of caspases is another critical hallmark feature of apoptosis. VCC-treated B1-a cells show an up-regulation of initiator caspase-9, involved in the mitochondria-mediated intrinsic apoptotic pathway, along with the effector caspase, caspase-3 (56, 59). However, caspase-8, which is a part of the extrinsic pathway, does not appear to be involved in the VCC-mediated apoptosis (56). Along with these, some other significant apoptotic features, such as flipping of phosphatidylserine and DNA fragmentation, have also been observed in the VCC-treated target cells (17, 59). VCC has been shown to trigger upregulation of caspases through the mitochondria-mediated activation pathway. Therefore, several studies explored the role of mitochondria in mediating the cytotoxic responses by VCC. One recent study has shown that VCC has a propensity to translocate to the mitochondria of the target cells via a clathrin-mediated pathway, leading to the mitochondrial membrane permeability transition, followed by the production of mitochondrial reactive oxygen species and the release of cytochrome c, both of which contribute to the VCC-mediated cell death (Fig. 2) (59).

*Ex vivo* studies in rabbit ileal loop have shown that VCC induces various pathological responses, including the recruitment of polymorphonuclear neutrophils, vascular alterations such as edema and dilation of lymphatic vessels, epithelial necrosis and apoptosis, and mucosal congestion. Collectively, these effects are very likely to compromise the intestinal barrier function, facilitating further infection and intensifying the disease progression (17). In the mouse model infected with *V. cholerae,* VCC has been shown to play crucial roles in establishing prolonged colonization in the small intestine and is primarily responsible for lethality. Interestingly, other major pathogenic factors like CT appear not to be necessary for the colonization or lethality. However, VCC does not appear to be responsible for affecting subsequent bacterial growth or contributing to bacterial spread to the spleen or liver in these models (7, 63–65).

The ability of the PFTs to elicit programmed cell death pathways has conventionally been attributed to their membrane-damaging pore-forming activity. This mechanism generally involves the free passage of ions across the plasma membranes through the pores formed by the PFTs, serving as a trigger for activating the apoptotic pathways in the target nucleated mammalian cells. However, a recent study employing the mutant variants of VCC, lacking the functional pore-forming capabilities, has shown that pore formation is not the essential requirement for eliciting apoptosis in the nucleated mammalian cells. It has been shown that the VCC mutants, having defective pore-forming ability, can evoke the characteristic features of apoptosis, such as increased cellular granularity, DNA fragmentation, membrane phosphatidylserine flipping, and caspase-3 activation, similar to those elicited by the wild-type toxin. These findings indicate that VCC can induce cell death even in the absence of the oligomeric pore assembly formation. Such pore-formation-independent cell death caused by VCC is presumably due to its ability to translocate to the mitochondria, causing mitochondrial damage, thus leading to cytochrome c release and subsequent activation of the apoptotic cascade involving caspases, ultimately resulting in cell death (Fig. 2) (59). These observations suggest that the cytotoxic activity of VCC persists even if the protein cannot form the functional β-barrel pores, indicating that its pore-formation ability may be less critical for affecting the nucleated cells in terms of cytotoxic responses. The dual functionality of VCC as a PFT to form transmembrane pores, and at the same time activating pore-formation-independent apoptotic pathways, raises questions about the exact significance of the pore-forming functionality. It remains an open question why a PFT, like VCC, would possess two distinct modes of killing of its target cells: (i) non-specific cell-killing through membrane damage due to its potent pore-forming activity, and (ii) cytotoxicity through activation of the specific programmed cell death pathway, which could be triggered even in the absence of the pore-forming activity. It is known that *V. cholerae* requires iron for its growth. It is possible that the VCC-induced pore-forming hemolytic activity against the erythrocytes would release hemoglobin from the lysed cells, and *V. cholerae* could utilize iron from such released hemoglobin for its growth. In contrast, programmed apoptotic cell death induced by VCC, even in the absence of the pore-forming activity, could be implicated in the tissue-damaging effects during the *V. cholerae* infection, and such damaging effects could be the means of accessing the nutrients by the pathogen. Furthermore, VCC-mediated programmed cell death of the immune cells, in particular, could be the immune evasion strategy of *V. cholerae*. Future studies would be required in this direction to explore and address such notions.

## INTERACTION OF VCC WITH THE IMMUNE CELLS

*V. cholerae* damages the gastrointestinal lining through the production of virulent factors in order to establish a pathogenic niche. As a virulence factor, VCC can cause cellular damage, attracting the immune cells to the site of infection. Therefore, the interaction of VCC with the immune cells may significantly impact the immune responses during the infections. Cells such as monocytes, neutrophils, and macrophages, along with other innate immune cells, are the first responders of the host immune system against the pathogen infection (66). This initial response can trigger the adaptive arm of the immune system to mount a more specific defense against the pathogen. Upon stimulation against the PFTs, receptors present on the innate immune cell surface, such as pattern recognition receptors (PRRs), Toll-like receptors (TLRs), and Nod-like receptors (NLRs), get activated. This activation initiates a downstream signaling cascade aimed at combating the infection (67).

### VCC activates pro-inflammatory responses in the innate immune cells

**Fig 3:**
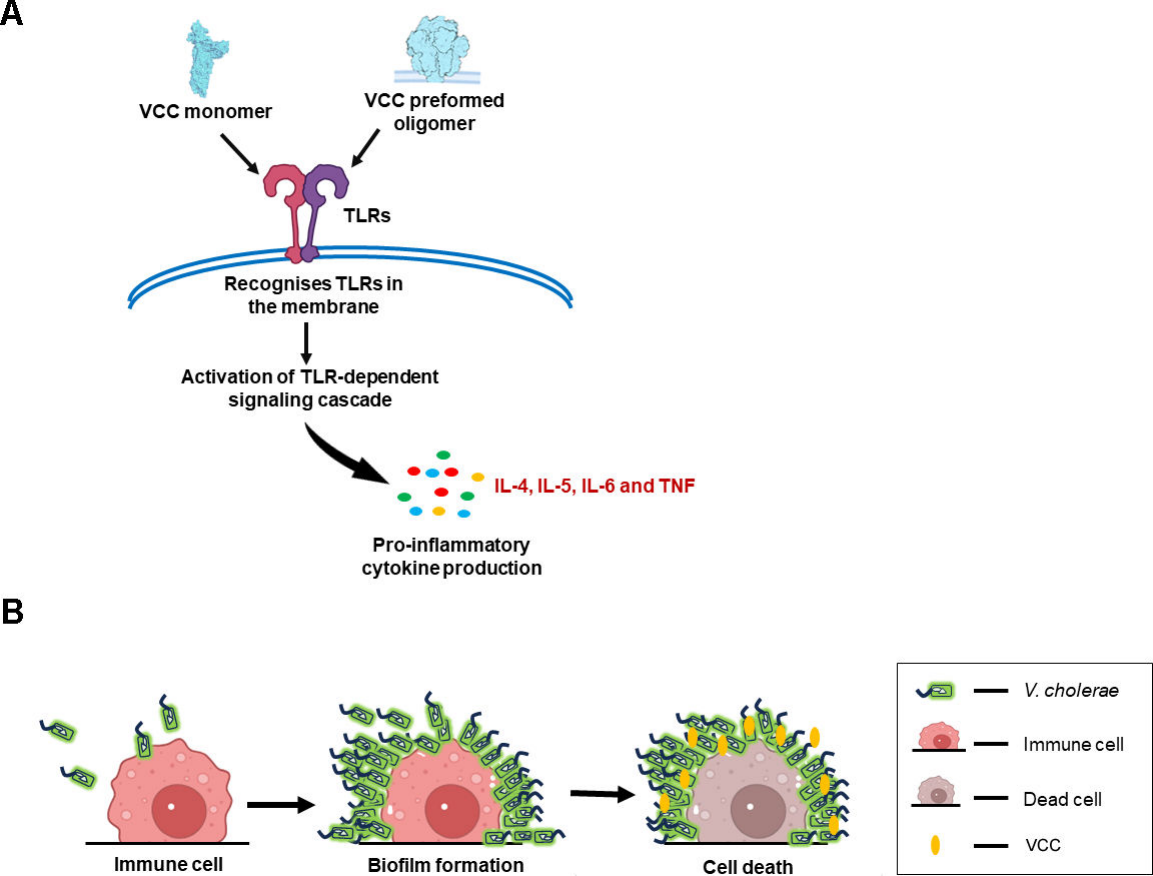
Interaction of VCC with the immune cells. (**A**) *Vibrio cholerae* interacts with intestinal epithelial cells during pathogenesis and subsequently engages intricately with the immune system, which recruits various immune cells, such as macrophages and dendritic cells. Pattern recognition receptors play a crucial role in recognizing VCC. TLRs can identify VCC in both its monomeric and membrane-bound oligomeric forms. This recognition triggers a TLR-mediated signaling cascade that results in the secretion of pro-inflammatory mediators, including IL-4, IL-5, IL-6, and TNF. (**B**) Biofilms are created by a group of microorganisms on a surface, serving as a protective shield against predation. *V. cholerae* forms biofilms on the surfaces of cells as a multicellular predation strategy to target immune cells. The biofilm formation on immune cells involves distinct phases, including attachment, growth into a three-dimensional structure, and finally detachment. This biofilm formation enables the pathogen to deliver its toxin, such as VCC, in high concentrations near the immune cell surface, thereby enhancing cell killing.

Generation of the pro-inflammatory responses is a critical response of the immune system against pathogens. Upon detecting a pathogen, immune cells recognize a plethora of pathogen-associated molecular patterns (PAMPs) and trigger the release of several pro-inflammatory mediators. PFTs such as VCC can act as a PAMP and can get recognized by the PRRs. Earlier studies have shown that the mast cells, which are one of the integral members of the innate immune system, get activated by VCC, leading to the secretion of cytokines such as IL-4, IL-5, IL-6, and TNF (60). Treatment of the B-1a cells with VCC has induced an increase in the TLR2 expression and activation of the mucosal immune response, such as the generation of IgA and IgM (56). A recent study shows that VCC engages a novel TLR1/4 heterodimer assembly and induces pro-inflammatory responses in the dendritic cells (68). In PerC macrophages, VCC can activate the apoptotic pathways mediated by caspases 9 and 7, with mitochondrial involvement (50). VCC can induce caspase 1-dependent inflammasome activation along with the secretion of cytokines that result in pyroptotic cell death (69). Interestingly, VCC intoxication of the innate immune cells appears to engage TLR-dependent signaling pathways, leading toward NF-κB activation and generation of pro-inflammatory responses (Fig. 3A) (50).

### Membrane-embedded VCC oligomers can trigger an immune response

Upon transition of VCC into the pore-forming oligomeric state, it does not revert to the monomeric form. This oligomeric form of VCC exhibits high resistance to denaturation and degradation by the proteases. Thus, upon cell death induction and membrane rupture by VCC, VCC oligomers, along with the membrane fragments, can act as the damage-associated molecular patterns to trigger an immune response (56, 70). VCC oligomers have been reported to activate the pro-inflammatory response in both monocytes and macrophages via TLR2/TLR6-mediated signaling cascades (70). Additionally, the oligomeric form of VCC elicits the upregulation of co-stimulatory molecules such as CD86 on the mouse macrophages (71). These observations suggest that the oligomeric assemblies of VCC are able to interact with and act on the immune cells without causing significant cytotoxicity (Fig. 3A).

### Role of VCC in biofilm formation

In environments where multiple pathogens coexist, there is always a chance of interacting with their own predator. Bacteria are subject to predation from the bacteriophages and from other bacterial species, which is considered to be one of the main causes of bacterial mortality (72, 73). Due to this continuous competition, bacteria have evolved various strategies to survive against predation. Apart from the single-cell strategies, such as escape from the immune system and the classical bacteriophage defense system, bacteria can collectively act by forming biofilms for protection against pathogens (74, 75). Studies have shown that in the aquatic environments, where *V. cholerae* exists both as planktonic and biofilm forms, planktonic bacteria are easily eliminated by the surface-feeding protozoa (76). This indicates that the biofilm-associated growth provides a suitable niche for bacterial survival. Beyond its conventional habitat in the aquatic environments, recent studies have highlighted the multicellular predation strategy of *V. cholerae* on human immune cells through biofilm formation (77). This biofilm formation occurs after breaching the epithelial barrier, and it affects various immune cell types, including NK cells, neutrophils, B cells, macrophages, and CD4^+^ T cells, but not monocytes (77).

Biofilm formation on the immune cell surfaces resembles the biofilm life cycle on abiotic surfaces and involves several phases: attachment, growth into a three-dimensional structure, and subsequent dispersal of bacteria (Fig. 3B) (78, 79). When macrophages were exposed to *V. cholerae* strains lacking the ability to form biofilms, there was a significant decrease in cell death, highlighting the importance of biofilm formation in pathogenesis (77). Furthermore, the deletion of the *hlyA* gene resulted in an even greater decrease in cell death. This biofilm formation on macrophages allows *V. cholerae* to enhance cell killing by releasing a high concentration of VCC near the macrophage surfaces. Unlike other bacteria that use biofilm formation as a refuge from the immune cells, *V. cholerae* employs this strategy as an aggressive multicellular strategy to attack the immune cells (77). These findings reveal how *V. cholerae* utilizes biofilm formation not merely for protection but also as a means of predation against host immune cells, in which VCC plays a pivotal role.

Altogether, VCC, as a pore-forming toxin, contributes enigmatic yet essential roles for the *V. cholerae* pathogenesis and host-pathogen interaction processes. Apart from the pore-forming function, VCC can induce cytotoxicity through activation of the programmed cell death pathways and can activate inflammatory immune responses. VCC has been shown to exhibit immune cell-killing effects in the biofilm environment formed by *V. cholerae* over the immune cells. VCC, even in the absence of the functional pore-formation ability, can induce cell death in its target cells. All these properties underscore a crucial role of VCC as an essential component of the *V. cholerae* virulence mechanism. However, further studies will be required to elucidate the intricate implications of VCC for the pathophysiological consequences during *V. cholerae* infection.

## CONCLUSION AND FUTURE PERSPECTIVES

Pathogenic bacteria employ PFTs as the major virulence factors, making these ancient cell-killing machineries an important target to combat bacterial pathogenesis. β-PFTs are a comparatively better-characterized group of bacterial PFTs. VCC is one of the most prominent β-PFT family members that has been studied extensively for its membrane pore-formation mechanism. Considerable numbers of studies have also described versatile pathophysiological properties of VCC that can modulate the outcome of the *V. cholerae* pathogenesis process. However, despite such efforts, the exact implication of VCC for the *V. cholerae* pathogenesis remains unclear. VCC is considered an accessory toxin of *V. cholerae*. Yet, VCC possesses multifaceted virulence functions. It remains to be explored why such an accessory toxin, which is not even a major virulence factor like CT, is retained by *V. cholerae*. It needs to be explored whether VCC assists in the bacterial physiological needs, such as iron acquisition, etc., or it imparts major roles in the pathogenesis process, or both. Answering the question, “how exactly the VCC-mediated membrane pore-formation and cytotoxicity are exploited by the cholera pathogen for its virulence purpose,” would possibly pave the way to design an alternate preventive/therapeutic strategy to combat *V. cholerae* pathogenesis.
